# Academic Goals and Attitudes Towards Institutional Authority: A Relationship Mediated by Life Satisfaction and Sense of Belonging to the School

**DOI:** 10.3390/ejihpe15050088

**Published:** 2025-05-19

**Authors:** Laura Giaquinto, Roberto Sanz, Pau García-Grau

**Affiliations:** 1 Doctoral School, Catholic University of Valencia, 46110 Valencia, Spain; laura.giaquinto@mail.ucv.com; 2 Faculty of Teaching and Educational Sciences, Catholic University of Valencia, 46110 Valencia, Spain; pau.garcia@ucv.es

**Keywords:** academic goals, life satisfaction, attitude towards institutional authority, sense of belonging to the school, school failure

## Abstract

Academic success is conditioned by different factors, related to academic goals, life satisfaction, and feelings of belonging to the school. This article aims to assess whether beliefs, attributions (academic goals), and feelings (life satisfaction and sense of belonging to the school) do or do not influence attitudes towards school authority (school, teachers, learning, norms, and families). A total of 510 students from six secondary schools participated and four questionnaires were used: academic goals, life satisfaction, attitude towards institutional authority, and sense of belonging to school. The focus was on the description of academic goals and negative attitudes towards authority, focusing on the mediating role of school belonging and life satisfaction. Descriptive, variance, and mediation analyses were conducted. The questionnaire with the highest scores was sense of belonging to school. The dimension with the least influence on negative attitudes within academic goals was social reinforcement. Males showed more negative attitudes towards authority, and an indirect and significant relationship was observed between academic goals and negative attitudes towards authority, mediated by life satisfaction and sense of belonging to school. In conclusion, the role of the school as a protective agent is confirmed. Therefore, intervening in negative attitudes towards authority would solve coexistence problems and increase life satisfaction, expectations of academic performance, and the feeling of belonging to the school.

## 1. Introduction

Academic success and/or failure at school can be conditioned by a variety of factors (Hilal et al., 2024). One of these has to do with motivation for learning (Ayllón-Salas et al., 2024). Also, and closely related to this, are the expectations that the individual may have for learning or, more generally, for the academic event. But academic goals, in turn, can also be influenced by a wide range of dimensions, of both a personal and social nature, which feedback on each other. Thus, feeling good about oneself (life satisfaction) and about the school, teachers, or classmates (sense of belonging to the school) can determine the way of acting and behaving towards school authority (attitude towards institutional authority).

Therefore, this research aims to delimit the relationships, direct or indirect, between all these variables, to establish a model that can explain all these relationships. To answer this objective, the following research questions are posed: can students’ academic goals explain their attitudes towards authority figures at school, and is this relationship mediated by the feeling of personal satisfaction and/or the feeling of belonging to the school?

The scientific literature has repeatedly addressed the issues of school failure and low academic goals in relation to academic performance (Boonk et al., 2018), as well as attitudes towards authority that affect the climate of coexistence and the academic performance of students. This study brings an interesting novelty. It aims to look at the relationship between the feeling of belonging to the school (Barrios et al., 2020; Yulianti et al., 2022) and the student’s own life satisfaction (Mitchall & Jaeger, 2018; Smith et al., 2022), which affects their emotions (Vera et al., 2021), with the construction of higher academic goals among students and with a more positive relationship towards authority figures. In this sense, we have not found any research that analyses the four variables jointly, examining their direct and indirect relationships. In the scientific literature, there is research that jointly analyses two of these variables, such as the studies by Carrasco and Luzón (2019) and John et al. (2018); these studies analyse the relationship between sense of belonging to the school and attitudes towards institutional authority. And there is the work by Valle et al. (2006), who looked for the dependence between academic goals and sense of belonging to the school. Also noteworthy is the research by Cava et al. (2013), which related attitudes towards institutional authority to students’ life satisfaction. Therefore, this research contributes a grain of sand to the scientific literature and, in addition, can contribute to improving academic performance and coexistence in schools, especially when the population comes from vulnerable neighbourhoods (Escarbajal et al., 2019; Mancila et al., 2024). The variables chosen are supported by the scientific literature (Rodríguez-Manteca & Rodríguez-Bravo, 2024) and form a theoretical model that needs to be corroborated.

Ultimately, the aim is to assess whether and how a set of beliefs, attributions (academic goals), and feelings (life satisfaction and sense of belonging to the school) do or do not influence attitudes towards school authority (school, teachers, learning, norms, and families).

### 1.1. Academic Goals and Attitude Towards Authority

Much research has analysed the causes, factors, and consequences of school failure and early school leaving. Unfortunately, both school failure and early dropout tend to affect, with greater incidence, the most vulnerable students, who have fewer resources and, therefore, fewer possibilities of reversing these situations. This research focuses on and analyses secondary schools located in the Valencian district of Poblados Marítimos, where a large number of young people are concentrated who are at risk of social exclusion due to family, social, and economic causes, as well as school failure, early school leaving and, therefore, the lack of adequate training and professional qualifications. In many cases, there are families with scarce economic resources, with a very high percentage of unemployment, with little prospect of employability for the youngest members of the family and, on many occasions, with jobs in the black economy on the fringes of legality or directly outside of it. Moreno et al. (2012) describe a series of factors that influence school failure and early school leaving, such as personal, attitudinal, and behavioural aspects, as well as educational and occupational factors. In addition, other variables can be added, such as family and cultural expectations, belief systems, and the type of social relationships that occur between peers. It is therefore obvious that many of these aspects are directly related to students’ academic goals and their attitude towards authority, understood as their relationship with teachers and their acceptance of school rules, as well as their perceived life satisfaction and sense of belonging to the school.

Academic goals can be defined as an integrated model or pattern of beliefs, attributions, affects, and feelings that direct students’ behavioural intentions. Therefore, they condition their affective, emotional, cognitive, and behavioural reactions in the school environment (Durán & Arias, 2015; Weiner, 1986). Along these lines, other studies associate these goals with motivation for learning (Barca et al., 2012). Thus, academic goals have a clear effect on academic performance, personal and pedagogical relationships with peers and teachers, the socioemotional bond with the school, and behaviour in the school environment (Valle et al., 2006).

One of these behaviours is evidenced, on the part of some students, in a transgressive and defiant attitude towards institutional authority (Cava et al., 2013), through provocation of teachers and parents, as well as non-compliance with school rules (Estévez & Emler, 2009; Estévez et al., 2011; Steffgen et al., 2013). These negative attitudes towards authority generate problems of coexistence in the classroom, disruptive behaviour, low expectations in terms of academic performance, low rates of life satisfaction among students (Gálvez et al., 2015), and a feeling of not belonging to the school.

### 1.2. The Importance of Life Satisfaction and Sense of Belonging to School in Shaping Academic Expectations

Life satisfaction is related to physical, cognitive, scholastic, emotional, and social indicators that help build the psychological strength and stability necessary to develop a quality life (Carranza et al., 2022; Garnique & Huanca, 2022; Higuita & Cardona, 2015). This can be conceived in terms of different dimensions or spheres, including family, school, social, and personal spheres (Chavarría & Barra, 2012). This research focuses on life satisfaction in the school environment, which can be defined as students’ satisfaction with their relationship with school, learning, teaching, and the feelings generated at school (Merino-Soto et al., 2017). Therefore, some studies (Salmela & Tuominen, 2010) relate, in adolescence, life satisfaction with academic performance, the presence of goals and/or expectations (Barrantes & Ureña, 2015; González-Peiteado et al., 2017), and the sense of belonging to the school (Jiao et al., 2017). Therefore, generating a good school climate, developing socioemotional competences in the classroom (Ruvalcaba et al., 2017), and creating positive relationships and attitudes between teachers and students, as well as establishing active methodologies, favours life satisfaction and improves academic expectations among students (Huayta et al., 2022; Leria & Salgado, 2019). Moreover, all this reduces the chances of early school leaving and school failure (Merino-Soto et al., 2017; Ventura-León et al., 2021).

Classical research (Finn, 1989; Spady, 1971; Tinto, 1975) has already linked a sense of belonging to school with improved academic results and the personal development of students. Currently, other research (Cordano, 2017; Rodríguez-Garcés et al., 2019) highlights its positive impact on school trajectory, life satisfaction, confidence, and predisposition for learning (Knekta et al., 2020). In addition, it reduces school dropout and failure and coexistence problems (Carrasco & Luzón, 2019; John et al., 2018). A sense of belonging is defined as the commitment and identification—cognitive and affective—of students with the educational institution. It has been shown that the absence of a sense of belonging to the school leads to attitudes of avoidance or rejection towards study (Corrales et al., 2016), thus decreasing the quality of academic goals and negatively influencing life satisfaction among adolescents (Moreta et al., 2017).

**Figure 1:**
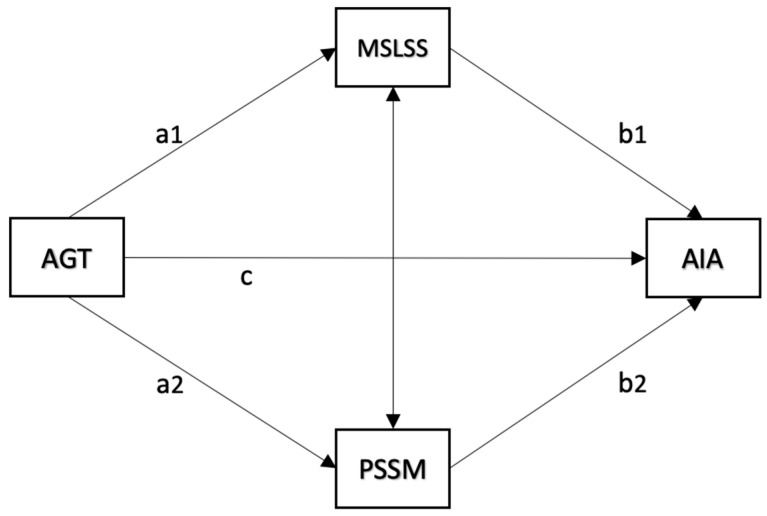
Hypothetical model of influence between variables. Note: AGT = achievement goal tendency; MSLSS = multidimensional students’ life satisfaction; PSSM = psychological sense of school membership; AIA = attitude towards institutional authority.

Therefore, the hypothetical model presented (Figure 1) describes a series of direct and indirect relationships between the variables analysed, based on the scientific literature. On the one hand, it is assumed that academic goals (AGT) directly influence attitude to authority (AIA). Thus, high academic goals—expectations of achieving a certain grade, being able to pursue a certain degree, etc.—lead to positive attitudes towards authority. In this sense, the student who pursues a specific objective is not interested in having problems with authority figures (teachers or principals).

On the other hand, we think that this direct relationship can also be mediated by two variables: life satisfaction (MSLSS) and sense of belonging to the school (PSSM). In this sense, the life satisfaction variable (MSLSS) has a direct relationship with students’ academic goals. When students feel good about themselves, they tend to set more ambitious academic goals. This affects their attitudes towards institutional authority. When students feel good about themselves, they tend not to create problems for themselves with other people, in this case with institutional authority. The variable sense of belonging to the school (PSSM) also has a direct relationship with students’ academic goals. When students feel that they are members of a community—the school—and involved with it, they tend to set more ambitious academic goals for themselves. Similarly, this affects their attitudes towards institutional authority. When students feel they are members of an institution—involved and participating—they tend not to create problems with institutional authority.

For all of the above, the present study has specific objectives, as follows:

(a) Describe the perception of the three types of goal orientations: learning goals (LGs), performance goals (PGs) and reinforcement goals (RGs), as well as their life satisfaction, attitude towards institutional authority and sense of school membership.
(b) Analyse the effect of gender and education level of parents on the scores in negative attitudes towards authority.
(c) Analyse the mediating effect of life satisfaction (MSLSS) and sense of school membership (PSSM) in the relationship between goal tendencies (AGT) and negative attitudes (AIA).

Considering these objectives, we stated the following hypotheses:

**H1.**  *Students with lower parental educational levels and male students show higher scores in negative attitudes towards authority (AIA).*

**H2.**  *Life satisfaction (MSLSS) and sense of school membership (PSSM) mediate the relationship between students’ goal orientations (AGT) and their negative attitudes towards institutional authority (AIA).*

## 2. Materials and Methods

### 2.1. Participants

A total of 510 students from six secondary schools in the city of Valencia, specifically from the Poblados Marítimos district, participated. The students were distributed between the third and fourth years of compulsory secondary education, with a fairly homogeneous distribution (third year = 51.7%). As shown in Table 1, male participants were significantly more represented: N = 271 (53.14%). Regarding the educational level of the fathers and mothers of the students, the most frequent category was university studies, both for the father (35.49%) and for the mother, in almost 42% of the cases, followed by secondary studies in both cases. The least frequent category was primary education in both cases (Table 1).

### 2.2. Instruments

Four different instruments were used. The first of them, a Spanish version of the achievement goal tendencies questionnaire (AGT) (Hayamizu & Weiner, 1991) proposed by González et al. (2000) was used. It is a 20-item Likert scale (from 1 = never to 5 = always) assessing the frequency of each statement. The instrument measures three types of goal orientations: learning goals (LGs), performance goals (PGs), and reinforcement goals (RGs). Previous studies with Spanish students found good levels of reliability and construct validity (González et al., 2000) and reliability (Veas et al., 2017). The original distribution of items proposed by previous studies was confirmed through confirmatory factor analysis in the present sample, obtaining satisfactory fit indices of the theoretical model to the data [χ^2^(df) = 767.566(167); χ^2^/df = 4.60; *p* < 0.001; CFI = 0.985; TLI = 0.983; IFI = 0.985; RMSEA = 0.04]. The internal consistency of the scores analysed in the present study was α = 0.89 and McDonald’s w of 0.89 for the learning goals/LG dimension; α = 0.79 and w = 0.82 for the social dimension (RG); and α = 0.90 and w = 0.91 for achievement goals (PGs). The total scale also showed high values for internal consistency of scores (α = 0.92 and ω = 0.90).

The second instrument is the multidimensional life satisfaction scale (MSLSS) (Huebner, 1994). It is a 13-item Likert scale (from 1 = never to 5 = always) assessing the frequency of each statement. For this study, the factors related to satisfaction with friends, satisfaction with families, and satisfaction with neighbourhood were eliminated as they were not the subject of this research. The fit of the original factor structure in the present sample had a good fit to the data in this study [χ^2^(df) = 264.295(63); χ^2^/df = 4.20; *p* < 0.001; CFI = 0.990; TLI = 0.988; IFI = 0.990; RMSEA = 0.04]. The internal consistency of the scores for the total scale analysed in the present study was α = 0.883 and ω = 0.893.

The third instrument is the attitude towards institutional authority scale (AIA) (Reicher & Emler, 1985). It is a 20-item Likert-type scale that assesses the degree of agreement or disagreement with the statements, using a scale of 1–5 (1 = do not agree at all and 4 = strongly agree). It consists of 4 dimensions: positive attitude towards school authority and school, positive attitude towards transgression, attitude/perception of injustice, and attitude undervaluing studies. However, for the present study, the overall score was used as a measure of general attitudes towards authority. Specifically, of the 20 items that make up the scale, only six items are formulated positively, measuring positive attitudes. These items were reversed to facilitate internal consistency and reliability analyses of the scale, showing adequate levels of internal consistency for the total score (α = 0.803 and ω = 0.821). With these scores reversed, the overall negative attitudes towards authority score was obtained and was similar to that of other studies, such as that of Cava et al. (2006), except that this study considered the entire scale. The original structure proposed by the authors presented a moderate fit to the data of the present study. However, item 7 contributed to a worsening of the scale’s internal consistency and reliability indices. After removing it, the fit of the original factor structure was satisfactory [χ^2^(df) = 577.365(146); χ^2^/df = 3.955, *p* < 0.001; CFI = 0.959; TLI = 0.952; IFI = 0.959; RMSEA = 0.049]. To answer the questions in this study, we considered using the scale total, which obtained internal consistency scores of α = 0.803 and ω = 0.821.

The fourth instrument is the psychological sense of school membership scale (PSSM) (Goodenow, 1993). It is an 18-item Likert scale (from 1 = never to 5 = always) assessing the frequency of each statement. The original theoretical model was shown to have a good fit to the data in this study [χ^2^(df) = 515.413(131); *p* < 0.001; χ^2^/df = 3.934; CFI = 0.963; TLI = 0.957; IFI = 0.963; RMSEA = 0.047]. The internal consistency of the total score scores in the present study was α = 0.848 and ω = 0.863.

### 2.3. Procedure

The questionnaires were answered in the schools themselves, voluntarily and anonymously, thanks to the collaboration of the school counsellors of the schools themselves. Permission and approval were requested from the Regional Ministry of Education, Culture, Universities and Employment of the Generalitat Valenciana, through the Autonomous Secretariat of Education, as well as from the headteachers of the participating schools. In the same way, authorisation was requested from the students’ families, as all of them were minors. Before answering the questionnaires, an explanatory video was shown, specifying the meaning and purpose of the research, as well as the procedure to be followed to answer the questionnaire. The students completed the questionnaires in the computer room under the supervision of the school counsellor, and, telematically, each student, with his/her assigned code, answered all the questions in the questionnaires. This took place over a period of one hour, so that all students in each school answered on the same day, during a 50 min class session. All schools completed the questionnaires within 3 weeks.

### 2.4. Data Analysis

Descriptive statistics were conducted using SPSS 25 software. We also analysed the impact of gender and the education level of parents on the students’ negative attitudes through *t*-tests and analysis of variance (ANOVA). Partial eta squared and Cohen’s d effect sizes were calculated. Eta squared values of 0.10, 0.25, and 0.37 and Cohen’s d values of 0.20, 0.50, and 0.80 were considered as small, medium, and large effect sizes, respectively (Goss-Sampson, 2020). Bonferroni correction was employed to avoid type I error in multiple testing.

Additionally, JASP software v.0.19.1 (JASP Team, 2024) was employed to estimate the internal consistency of the scores and to specify and test the fit of the theoretical model to the data through a structural equation model (SEM). We examined the effect of AGT on AIA and the mediating influence of MSLSS and PSSM in this relationship (see Figure 1). The AGT latent variable included the observed factor scores. Indirect and total direct effects were calculated for these relationships. Path coefficients—regression coefficients—ranged from −1 to +1.

To evaluate the model’s fit to the data, we used the following indicators: the comparative fit index (CFI), incremental fit index (IFI), Tucker–Lewis index (TLI), and the root mean square error of approximation (RMSEA). A non-significant chi-square test indicated an adequate fit between the specified model and the observed data. Good model fit was further evidenced by RMSEA values close to zero (Browne & Cudeck, 1993) and CFI, IFI, and TLI values approaching 1 (MacCallum & Austin, 2000; Kline, 2023).

To assess the statistical power, a post hoc calculation given a desired alpha of 0.05 and 510 participants using G*Power 3 (Faul et al., 2007) was employed. A medium effect size was selected for the model. The number of predictors was nine predictors (three of them belonging to the AGT latent variable and six to the mediation model). The statistics showed strong values [1- = 0.99, Critical F(9, 500) = 1898, *p* = 0.05, f2 = 0.15, non-centrality parameter λ = 76.50].

## 3. Results

### 3.1. Specific Objective 1: Descriptive Results

Regarding the AGT scale factors and the total score, the variability in the scores obtained is noteworthy. While the performance (PG) dimension (M = 4.29 SD = 0.87) stands out as the highest factor in the mean scores, the reinforcement (RG) factor (M = 2.39, SD = 0.91) is the lowest, which reaffirms the lower relevance of social pressures or motivations (Table 2). On the other hand, in the total scale the mean is 3.21 (SD = 0.68), which represents a tendency towards neutral–high in the general perception of academic goals.

Regarding the sense of school membership, it was the construct with a higher average score (M = 3.59, SD = 0.59), followed by the multidimensional life satisfaction overall score, where the average score was 3.13 (SD = 0.73), and negative attitudes (M = 2.55, SD = 0.53). Normality analysis indicated acceptable distributional values for all variables. One variable showed moderate skewness (−1.74) and kurtosis (3.11), but these values are within acceptable limits given the large sample size (N = 510), suggesting that the use of parametric methods was appropriate (Table 2).

### 3.2. Specific Objective 2: Score Differences According to Gender and Parents Education Level

We then analysed the gender differences in negative attitudes. The results of the *t*-test showed statistically significant differences with small effect sizes (t = 3.701, *p* < 0.001, d = 0.329). This result indicated that male students (M = 2.63, SD = 0.53) scored higher in negative attitudes than female students (M = 2.46, SD = 0.51). In addition, an ANOVA was performed to analyse differences in AIA based on parents’ education levels. The results revealed no statistically significant differences in negative attitudes in the case of the father’s education level [F(2, 398) = 1.96, *p* > 0.05, η^2^_p_ = 0.010] or the mother’s education level [F(2, 438) = 1.09, *p* > 0.05, η^2^_p_ = 0.05], suggesting that parental education level does not influence students’ scores in AIA. In that sense, these results partially confirm H1. On the one hand, the relationship between students’ gender and negative attitudes towards authority was confirmed. On the other hand, no differences were found between the parents’ level of education and negative attitudes towards authority; thus, the second statement of H1 cannot be confirmed.

### 3.3. Specific Objective 3: Mediation Analysis

Next, we examined the effect of AGT on AIA and the mediating influence of MSLSS and PSSM on this relationship. As a first step, we analysed the Pearson correlations between the variables of the model. The AGT overall score also positively correlated with overall life satisfaction (MSLSS; r = 0.481; *p* < 0.001) and perceived school membership (PSSM; r = 0.335; *p* < 0.001) and negatively associated with attitudes towards institutional authority (AIA; r = –0.309; *p* < 0.001). These results confirm H2. Therefore, the mediation of the variables life satisfaction and sense of belonging to the school in the relationship between academic goals and negative attitude towards authority is confirmed.

The reinforcement goals showed non-significant correlations with school membership (PSSM; r = −0.002; *p* > 0.05) and negative attitudes towards authority (AIA; r = −0.002; *p* > 0.05). All the external scales (MSLSS, AIA, PSSM) correlated significantly with one another, in expected directions, with the strongest being the negative relation between school satisfaction and academic amotivation (r = −0.640; *p* < 0.001). The correlations among MSLSS and PSSM were also positive and highly significant, indicating that the higher the life satisfaction, the greater sense of school membership. The correlations between both MSLSS and PSSM with AIA were negative and statistically significant (r = −0.640; *p* < 0.001 and r = −0.613; *p* < 0.001, respectively), indicating that higher life satisfaction and a stronger sense of school membership were associated with less negative attitudes toward authority. As expected, all the AGT factors were highly correlated with each other in a positive way and with the overall AGT score (*p* < 0.001 in all cases) (Table 3).

Next, we tested our theoretical model. The model indicated values close to an adequate fit to the data (χ^2^ = 45.52, df = 6, χ^2^/df = 7.58, *p* < 0.001), with additional fit indices of [CFI = 0.962, IFI = 0.962, TLI = 0.905, SRMR = 0.070, RMSEA = 0.11(0.80–0.14)]. However, the error and χ^2^/df indicators could be improved through the exclusion of covariance residuals between the RG and PG domains, as suggested by the modification index analysis. This re-specification resulted in lower error model fit values and a satisfactory overall model fit (χ^2^ = 23.773, df = 5, χ^2^/df = 4.75, *p* < 0.001), indicating an acceptable fit under 5.00 (Schumacker & Lomax, 2004). Furthermore, additional fit indices showed satisfactory fit values [CFI = 0.982, IFI = 0.962, TLI = 0.946, SRMR = 0.050, RMSEA = 0.08(0.05–0.12)].

First, the factor loadings of the indicators of the AGT latent variable showed statistically significant factor loadings, indicating that their contribution to the latent variable was relevant (Table 4). In addition, the factor variances indicated that the factor is significantly capturing the latent construct (b = 0.524, z = 8.058, *p* < 0.001, CI [0.397, 0.652]), demonstrating that the latent factor is consistent and relevant for the sample studied.

The regression coefficients showed that the direct effect from AGT to AIA was not statistically significant (b = −0.042, z = −0.961, *p* > 0.05, CI [−0.127, 0.043]), indicating that, by itself, AGT did not predict changes in AIA (attitudes).

Regarding the influence of AGT on the mediators, the prediction of MSLSS was statistically significant and positive (b = 0.653, z = 9.474, *p* < 0.001, CI [0.518, 0.788]), indicating that the higher the score in AGT, the greater the perceptions of MSLSS. Likewise, the prediction of the second mediator (PSSM) was also statistically significant and positive (b = 0.389, z = 7.670, *p* < 0.001, CI [0.290, 0.489]), meaning that the higher AGT scores predict greater sense of belonging.

The influence of both mediators also significantly predicted the outcome (AIA—attitudes). The path coefficients showed that higher PSSM scores predicted lower scores in AIA (negative attitudes) (b = −0.304, z = 7.615, *p* < 0.001, CI [−0.382, −0.225]). This negative influence was also found in the regression from MSLSS to AIA, which revealed a negative and noteworthy prediction of negative attitudes (b = −0.280, z = −6.558, *p* < 0.001, CI [−0.364, −0.196]).

Regarding the parameters defined in the model to assess the mediation effects, Table 5 presents the defined parameters of the SEM model, highlighting the indirect effects, the total indirect effect, and the total effect of the relationship between AGT and AIA. The results indicated that both mediators played an important role and showed statistically significant indirect effects on AIA across both pathways (AGT → PSSM → AIA; b = −0.118, z = −5.468, *p* < 0.001) and (AGT → MSLSS → AIA; b = −0.183, z = −0.540, *p* < 0.001). Both relationships were negative, indicating that higher AGT scores predict higher perceptions of PSSM and MSLSS, and this produces lower AIA scores, indicating the prediction of lower negative attitudes.

The total indirect effect, indicating the joint effect of both mediators on the relationship between AGT and attitudes, was also significant (b = −0.301, z = −7.695, *p* < 0.001), indicating that both PSSM and MSLSS jointly and inversely mediate the relationship between AGT and AIA, following the same pattern as above.

Finally, the total effect (sum of direct + indirect effects) of AGT on AIA is also negative and significant (b = −0.343, z = −7.566, *p* < 0.001). This confirms that AGT globally predicts lower AIA scores, although this impact is mainly mediated by PSSM and MSLSS. In other words, the impact of AGT on AIA is largely indirect.

The residual variances were in all cases statistically significant (*p* < 0.001), indicating that the latent factor explains much of the indicator variability and, therefore, that the model fits correctly for each indicator.

**Figure 2:**
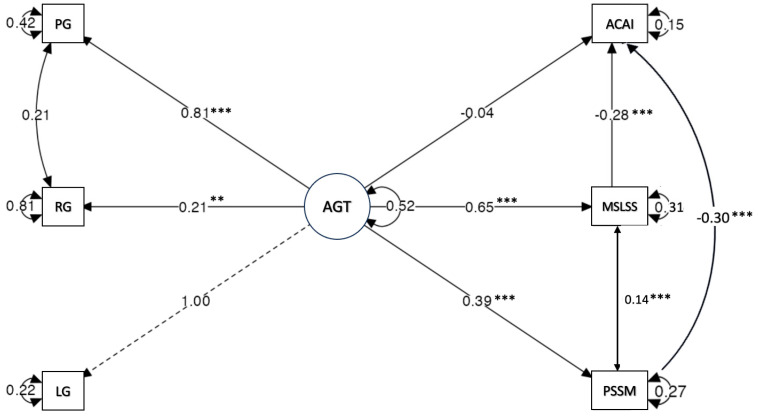
Summary plot of the SEM path coefficients. Note: PG = performance goals; RG = reinforcement goals; LG = learning goals; AIA = attitudes towards institutional authority; AGT = achievement goal tendency; MSLSS = multidimensional students’ life satisfaction; PSSM = psychological sense of school membership. ** *p* < 0.01; *** *p* < 0.001.

Overall, the proportion of variance explained by the model for each indicator showed that the LG (R^2^ = 0.703), PG (R^2^ = 0.450), MSLSS (R^2^ = 0.419), and AIA (R^2^ = 0.481) dimensions were adequately explained by the model. To a lesser extent, PSSM (R^2^ = 0.229) and RG (R^2^ = 0.028) indicated a higher variance, not explained by the model, than the rest, and contributed the least overall (Figure 2 and Table 6).

## 4. Discussion

The aim of the article was to assess what beliefs, attributions (academic goals), and feelings (life satisfaction and sense of belonging to the school) have an influence and how they influence students’ attitudes towards authority.

After describing the results obtained in the different questionnaires and analysing the direct and indirect relationships between them, it is relevant to assess whether or not these results coincide with similar research. It is therefore a matter of discussing and comparing with other realities, circumstances, and/or times. Furthermore, in the discussion of the results we will be able to verify the degree to which the hypotheses set out at the beginning of this research have been fulfilled or not.

Overall, the questionnaire with the highest mean scores is the sense of belonging to school (PSSMS), followed by life satisfaction (MSLSS), academic goals (AGT) and, finally, negative attitudes towards institutional authority (AIA). These results may be quite consistent with the reality of the students participating in this research. For them, the school can become a protective factor against their social and family reality, hence their commitment to the school institution. It should not be forgotten that a high percentage of the participating students are at risk of social exclusion and have very significant family and social deprivations. However, in studies with children and adolescents, in general, it is observed that the lowest life satisfaction scores are related to the school environment (Chavarría & Barra, 2012; Oyanedel et al., 2015), probably because for this population, school is a more formal institution with more rigid rules. Therefore, as Luckner and Pianta (2011) and Albornoz and Cornejo (2017) state, if teachers show a close and caring relationship with these students at risk of social exclusion, they will develop positive attitudes and commitment to the educational space. In this way, teachers can prevent or remedy negative social climates (Aron et al., 2012; Cava et al., 2013). It is also logical, due to the characteristics of the sample, that they present negative attitudes towards institutional authority figures. Poor academic results, lack of protective factors, lack of mechanisms and skills for conflict resolution, etc., lead them to a defiant, even transgressive attitude towards institutional authority and the rules of behaviour and functioning of the school (Cava et al., 2010; Estévez et al., 2011; Hilal et al., 2024).

Within the academic goals questionnaire, the dimension with the highest mean scores is performance (PG), while the dimension with the lowest mean score is social reinforcement (RG). These results contradict those obtained by Valle et al. (2006), where it is stated that the search for the approval of others—social evaluation by peers—directs academic goals, above the achievement of good grades. In terms of negative attitudes towards institutional authority, male students show more negative attitudes than female students. These data contradict the results obtained by Cava et al. (2013), with Spanish and Mexican students, where no gender differences were found. On the other hand, no differences were found according to the level of education of fathers and mothers. The results of these studies contradict our initial H1, as they do not find differences according to either gender or the parents’ level of education. In our research, differences are only found according to the gender of the students, which partly confirms H1.

Regarding the effects of academic goals (AGT) on negative attitudes towards authority (AIA), under the mediating influence of life satisfaction (MSLSS) and sense of belonging to school (PSSMS), the following results are observed. On the one hand, there is no direct and significant relationship between academic goals and negative attitudes towards authority. This partly contradicts the findings of Steinmayr and Spinath (2009), who related students’ academic goals to a commitment to learning, which allowed them to generate closer and more positive relationships (feelings, attitudes, and behaviours) with school authority figures (teachers). However, an indirect—mediated—and significant relationship does appear when the variables life satisfaction and sense of belonging to the school intervene. In both cases, it is observed that the higher the mean scores in academic goals, the higher the mean scores in life satisfaction and sense of belonging to the school and, in turn, the lower the mean scores in negative attitudes towards authority. Research by Carrasco and Luzón (2019) and Villalobos et al. (2016) go in the same direction. This research also confirms H2. Thus, the mediated relationship between academic goals and negative attitude towards authority can be confirmed by the variables of life satisfaction and sense of belonging to the school. Cava et al. (2006) also state that adolescents who show a negative attitude towards authority have low rates of life satisfaction and integration problems in the classroom. On the other hand, higher mean scores in academic goals, higher mean scores in life satisfaction. These results coincide with those obtained by Alfaro et al. (2016) and by Moreta et al. (2017) with Ecuadorian university students. Similarly, higher mean scores in academic goals lead to higher mean scores in sense of belonging to school. These results coincide with those obtained by Rodríguez-Garcés et al. (2019) with Chilean elementary and secondary school students and with those of Orellana and Segovia (2014).

## 5. Conclusions

After analysing the relationships between academic goals, life satisfaction, the feeling of belonging to the school, and attitudes towards institutional authority, in a group of students in a situation of vulnerability and at clear risk of social exclusion, the following can be concluded.

The role of the school as a protective agent is evident, as it can help to improve students’ expectations regarding their academic goals, which results in greater personal satisfaction and a greater sense of belonging to the school. This leads to a more positive attitude towards institutional authority, which helps to improve later life development. Therefore, preventing and intervening in disruptive behaviour associated with negative attitudes towards institutional authority would help to solve problems of coexistence in the classroom and in the school, increase the indices of life satisfaction among students, increase expectations in terms of academic performance, and improve the feeling of belonging to the school.

It can also be observed that men have a more negative attitude towards institutional authority. On the other hand, no differences are observed according to the parents’ level of studies. Curiously, in terms of academic goals, the social influence of the peer group has the least impact on academic performance (grades). This should lead us to consider pedagogical measures—such as tutoring, support classes, etc.—for these students, in order to improve their academic performance. The improvement in academic performance, as has been demonstrated and would result in an improvement in the indices of life satisfaction, sense of belonging to school, and attitudes towards institutional authority.

## 6. Theorical and Practical Implications

These results have direct implications for schools and for policy makers in charge of educational issues. On the one hand, schools need to foster students’ emotional attachment to their own school (Guzmán et al., 2025). They must make the school feel like their own, generating activities and actions that enhance attachment and a sense of belonging and security. Similarly, teachers should become ‘resilience tutors’ (Sanz et al., 2023; Serrano et al., 2023), providing support, security, and confidence to students. Academic support, the provision of resources and psycho-pedagogical attention for this group is fundamental to improve and increase academic expectations, which favours greater personal satisfaction and a greater sense of belonging and improves attitudes towards institutional authority. Some of the practical measures that could be implemented include, on the one hand, opening schools outside school hours so that students could use the sports facilities or academic material—computers, library, etc.—in order to enhance the feeling of belonging. It would also be interesting to implement workshops to improve self-esteem and self-concept, thus improving students’ life satisfaction, and to build self-confidence in the role of the school as a caring and protective agency and to develop positive relationships between students and teachers, e.g., through sport. All these actions will result in better academic achievement and a more positive attitude towards authority.

As for the limitations of this article, the following stand out. On the one hand, due to the fact that it deals with a specific group, in a specific area, the results obtained cannot be generalised to the population as a whole. On the other hand, it should be noted that both social desirability bias and recall bias may influence the research presented. Social desirability bias refers to the tendency of participants to respond in a way that they believe will be viewed favourably by others, while recall bias occurs when the way a person remembers a past event is influenced by their current state. As for future lines of research, these concern the impact of all these questionnaires on the variable risk of school failure and early school leaving, which will allow us to identify the typology of students at risk and to develop more effective and efficient intervention programs to deal with school failure, allowing us to act in response to the specific needs of each student.

## Figures and Tables

**Table 1:** Characteristics of participants.

	N	%
**Centre**		
Cabanyal	19	3.725
Isabel de Villena	47	9.216
El Grao	42	8.235
Distrito Marítimo	160	31.373
Baleares	53	10.392
Serpis	189	37.059
Total	510	100
**Gender**		
Male	271	53.137
Female	239	46.863
Total	510	100
**Course**		
Third Secondary	264	51.765
Fourth Secondary	246	48.235
Total	510	100
**Father’s Educ. level**		
Primary	42	8.23
Secondary	178	34.90
University	181	35.49
Missing	109	21.37
Total	510	100
**Mother’s Educ. level**		
Primary	44	8.63
Secondary	183	35.88
University	214	41.96
Missing	69	13.53
Total	510	100

**Table 2:** Descriptive statistics of the scores in AGT, MSLSS, PSSM, and AIA.

Abbreviated Version of the Item	M	SD	Min	Max	Skewness	Kurtosis
Learning Goals	3.02	0.86	1.00	5.00	−0.152	−0.152
Reinforcement Goals	2.39	0.91	1.00	4.83	−0.538	−0.538
Performance Goals	4.29	0.87	1.00	5.00	0.509	0.509
Overall AGT score	3.21	0.68	1.00	4.80	−0.419	−0.419
Overall MSLSS score	3.134	0.731	1.08	4.92	−1.737	−1.737
Overall PSSM score	3.586	0.589	1.89	5.00	3.112	3.112
Negative Attitudes (AIA)	2.548	0.530	1.16	4.00	−0.652	−0.652

**Table 3:** Pearson correlations between the scores in MSLSS, PSSM, AIA, and AGT overall score and its factors.

Variable	1	2	3	4	5	6	7
1. AGT_Learning Goals	—						
2. AGT_Reinforcement Goals	0.287 ***	—					
3. AGT_Performance Goals	0.507 ***	0.376 ***	—				
4. Overall AGT score	0.818 ***	0.693 ***	0.793 ***	—			
5. Overall MSLSS score	0.547 ***	0.093 *	0.433 ***	0.481 ***	—		
6. Overall AIA score	−0.381 ***	0.059	−0.363 ***	−0.309 ***	−0.640 ***	—	
7. Overall PSSM score	0.408 ***	−0.002	0.334 ***	0.335 ***	0.644 ***	−0.613 ***	—

*Note*: * *p* < 0.05, *** *p* < 0.001.

**Table 4:** Factor loadings of the latent variable.

						95% CI	
Latent	Indicator	Estimate	SE	z	*p*	Lower	Upper
**AGT**	LG	1.000	0.000	—	—	1.000	1.000
	RG	0.211	0.073	2.877	0.004	0.067	0.354
	PG	0.807	0.083	9.739	<0.001	0.645	0.970

*Note*: CI = confidence interval; SE = standard error; LG = learning goals; RG = reinforcement goals; PG = performance goals.

**Table 5:** Indirect and total effects of the SEM model.

					95% CI	
Type of Effect	Estimate	SE	z	*p*	Lower	Upper
Indirect 1 AGT → PSSM → AIA	−0.118	0.022	−5.468	<0.001	−0.161	−0.076
Indirect 2 AGT → MSLSS → AIA	−0.183	0.033	−5.540	<0.001	−0.247	−0.118
Indirect total effect	−0.301	0.039	−7.695	<0.001	−0.378	−0.224
Total effect	−0.343	0.045	−7.566	<0.001	−0.431	−0.254

**Table 6:** Summary of the main results.

Men have a more negative attitude towards authority than women
Parents’ level of education shows no difference in negative attitudes towards children’s authority
**Higher** AGT (Academic Goals) = **Higher** MSLSS (Life Satisfaction)
**Higher** AGT (Academic Goals) = **Higher** PSSM (Sense of Belonging to School)
**Higher** MSLSS (Life Satisfaction) = **Lower** AIA (Negative Attitudes Towards Authority)
**Higher** PSSM (Sense of Belonging to the School) = **Lower** AIA (Negative Attitudes Towards Authority)
**Higher** AGT (Academic Goals) = **Higher** MSLSS (Life Satisfaction) = **Higher** PSSM (Sense of Belonging to School) = **Lower** AIA (Negative Attitudes Towards Authority)

## Data Availability

The raw data supporting the conclusions of this article will be made available by the authors on request.

## References

[B1-ejihpe-15-00088] Albornoz N., Cornejo R. (2017). Discursos docentes sobre el vínculo con sus estudiantes: Tensiones, enfrentamiento y distancia. Estudios Pedagógicos.

[B2-ejihpe-15-00088] Alfaro J., Guzmán J., Reyes F., García C., Varela J., Sirlopú D. (2016). Satisfacción global con la vida y satisfacción escolar en estudiantes chilenos. Psykhe.

[B3-ejihpe-15-00088] Aron A. M., Milicic N., Armijo I. (2012). Clima social escolar: Una escala de evaluación-Escala de Clima Social Escolar, ECLIS. Universitas Psychologica.

[B4-ejihpe-15-00088] Ayllón-Salas P., Fernández-Martín F. D., Arco-Tirado J. L. (2024). Grit as a predictor of self-regulated learning in students at risk of social exclusion. Curr Psychol.

[B5-ejihpe-15-00088] Barca A., Almeida L., Porto A., Peralbo M., Brenlla J. (2012). Motivación escolar y rendimiento: Impacto de metas académicas, de estrategias de aprendizaje y autoeficacia. Anales de Psicología.

[B6-ejihpe-15-00088] Barrantes K., Ureña P. (2015). Bienestar psicológico y bienestar subjetivo en estudiantes universitarios costarricenses. Revista Intercontinental de Psicología y Educación.

[B7-ejihpe-15-00088] Barrios Y. B., Narváez M. A., Landazabal M. S. C., Vargas L. E. (2020). Clima organizacional de los procesos de participación comunitaria de una institución educativa. Caso estudio. Utopía y Praxis Latinoamericana.

[B8-ejihpe-15-00088] Boonk L., Gijselaers H., Ritzen H., Brand-Gruwel S. (2018). A review of the relationship between parental involvement indicators and academic achievement. Educational Research Review.

[B9-ejihpe-15-00088] Browne M. W., Cudeck R., Bollen K., Long J. (1993). Alternative ways of assessing model fit. Testing structural equation models.

[B10-ejihpe-15-00088] Carranza R., Mamani O., Castillo R., Lingan S., Cabrera I. (2022). Influence of family and academic satisfaction on life satisfaction among Peruvian university students: The mediating role of self-esteem. Frontiers in Education.

[B11-ejihpe-15-00088] Carrasco C., Luzón A. (2019). Respeto docente y convivencia escolar: Significados y estrategias en escuelas chilenas. Psicoperspectivas.

[B12-ejihpe-15-00088] Cava M. J., Buelga S., Musitu G., Murgui S. (2010). Violencia escolar entre adolescentes y sus implicaciones en el ajuste psicosocial: Un estudio longitudinal. Revista de Psicodidáctica.

[B13-ejihpe-15-00088] Cava M. J., Estévez E., Buelga S., Musitu G. (2013). Propiedades psicométricas de la Escala de Actitudes hacia la Autoridad Institucional en adolescentes (AAI-A). Anales de Psicología.

[B14-ejihpe-15-00088] Cava M. J., Musitu G., Murgui S. (2006). Familia y violencia escolar: El rol mediador de la autoestima y la actitud hacia la autoridad institucional. Psicothema.

[B15-ejihpe-15-00088] Chavarría M. P., Barra E. (2012). Life satisfaction in adolescents: Relationship with self-efficacy and perceived social support. Terapia Psicológica.

[B16-ejihpe-15-00088] Cordano M. (2017). El sentido de pertenencia al colegio es un factor clave en el bienestar de los alumnos.

[B17-ejihpe-15-00088] Corrales T., Waterford M., Goodwin-Smith I., Wood L., Yourell T., Ho C. (2016). Childhood adversity, sense of belonging and psychosocial outcomes in emerging adulthood: A test of mediated pathways. Children and Youth Services Review.

[B18-ejihpe-15-00088] Durán E., Arias D. (2015). Validez del Cuestionario de Metas Académicas (CMA) en una muestra de estudiantes universitarios. Cuadernos Hispanoamericanos de Psicología.

[B19-ejihpe-15-00088] Escarbajal A., Esssomba M. A., Abenza B. (2019). El rendimiento académico de los alumnos de la ESO en un contexto vulnerable y multicultural. Educar.

[B20-ejihpe-15-00088] Estévez E., Emler N., Larson J. E. (2009). Individual differences in attitude to school and social reputation among peers: Implications for behavioral adjustment in educational settings. Educational psychology.

[B21-ejihpe-15-00088] Estévez E., Jiménez T., Moreno D. (2011). Cuando las víctimas de violencia escolar se convierten en agresores: “¿Quién va a defenderme?”. European Journal of Education and Psychology.

[B22-ejihpe-15-00088] Faul F., Erdfelder E., Lang A. G., Buchner A. (2007). G*Power 3: A flexible statistical power analysis program for the social, behavioral, and biomedical sciences. Behavior Research Methods.

[B23-ejihpe-15-00088] Finn J. (1989). Withdrawing from school. Review of Educational Research.

[B24-ejihpe-15-00088] Garnique R., Huanca O. (2022). Autoestima y satisfacción familiar como predictores de la satisfacción con la vida en universitarios de la sierra peruana. Tesis de Maestría.

[B25-ejihpe-15-00088] Gálvez J. L., Vera D., Trizano I. (2015). Estudio confirmatorio del cuestionario para evaluar el clima social del centro escolar en Chile. Revista Mexicana de Psicología.

[B26-ejihpe-15-00088] González C., Navas L., Torregrosa G., Marchena E., Alcalde C. (2000). Las metas en situación de aprendizaje: Un análisis en Primaria y Secundaria Obligatoria. La perspectiva de la educación en el siglo que empieza.

[B27-ejihpe-15-00088] González-Peiteado M., Pino M., Penado M. (2017). Estudio de la satisfacción percibida por los estudiantes de la UNED con su vida universitaria. RIED: Revista Iberoamericana de Educación a Distancia.

[B28-ejihpe-15-00088] Goodenow C. (1993). Classroom belonging among early adolescent students: Relationships to motivation and achievement. The Journal of Early Adolescence.

[B29-ejihpe-15-00088] Goss-Sampson M. A. (2020). Statistical analysis in JASP.

[B30-ejihpe-15-00088] Guzmán C., Schoeps K., Montoya-Castilla I., Gil-Gómez J.-A. (2025). Impacto de la inteligencia emocional y del clima escolar sobre el bienestar subjetivo y los síntomas emocionales en la adolescencia. Estudios Sobre Educación, Early Access.

[B31-ejihpe-15-00088] Hayamizu T., Weiner B. (1991). A test of Dweck’s model of achievenement goal to perceptions of ability. Journal of Experimental Education.

[B32-ejihpe-15-00088] Higuita L. F., Cardona J. A. (2015). Concepto de calidad de vida en la adolescencia: Una revisión crítica de la literatura. CES Psicología.

[B33-ejihpe-15-00088] Hilal M., Khabbache H., Ait Ali D. (2024). Dropping out of school: A psychosocial approach. Advances in Medicine, Psychology, and Public Health.

[B34-ejihpe-15-00088] Huayta M., Turpo J., Mamani O. (2022). Predictores de la satisfacción con los estudios en universitarios de salud durante la pandemia COVID-19. Revista Cubana de Enfermería.

[B35-ejihpe-15-00088] Huebner E. S. (1994). Preliminary development and validation of a multidimensional life satisfaction scale for children. Psychological Assessment.

[B36-ejihpe-15-00088] JASP Team (2024). JASP (Version 0.19.1) *[Computer software]*.

[B37-ejihpe-15-00088] Jiao C., Wang T., Liu J., Wu H., Cui F., Peng X. (2017). Using exponential random graph models to analyze the character of peer relationship networks and their effects on the subjective well-being of adolescents. Frontiers in Psychology.

[B38-ejihpe-15-00088] John E., Daun N., Moronski K. (2018). Public policy and higher education: Reframing strategies for preparation, access, and college success.

[B39-ejihpe-15-00088] Kline R. B. (2023). Principles and practice of structural equation modeling.

[B40-ejihpe-15-00088] Knekta E., Chatzikyriakidou K., McCartney M. (2020). Measuring university students’ interest in biology: Evaluation of an instrument targeting Hidi and Renninger’s individual interest. International Journal of STEM Education.

[B41-ejihpe-15-00088] Leria F. J., Salgado J. A. (2019). Efecto del clima social escolar en la satisfacción con la vida en estudiantes de primaria y secundaria. Revista de Educación.

[B42-ejihpe-15-00088] Luckner A., Pianta R. (2011). Teacher-student interaction in fifth grade classrooms: Relation with children’s peer behavior. Journal of Applied Developmental Psychology.

[B43-ejihpe-15-00088] MacCallum R. C., Austin J. T. (2000). Applications of structural equation modeling in psychological research. Annual Review of Psychology.

[B44-ejihpe-15-00088] Mancila I., Márquez M. J., Redón S., Angulo J. F. (2024). Procesos de abandono escolar en España y Chile. La visión de los adolescentes más vulnerables. Revista Complutense de Educación.

[B45-ejihpe-15-00088] Merino-Soto C., Domínguez S., Fernández M. (2017). Validación inicial de una Escala Breve de Satisfacción con los Estudios en estudiantes universitarios de Lima. Educación Médica.

[B46-ejihpe-15-00088] Mitchall A. M., Jaeger A. J. (2018). Parental influences on low-income, first-generation students’ motivation on the path to college. The Journal of Higher Education.

[B47-ejihpe-15-00088] Moreno A., López A., Segado S. (2012). La transición de los jóvenes a la vida adulta. Crisis económica y emancipación tardía. Colección Estudios Sociales.

[B48-ejihpe-15-00088] Moreta R., Gabior I., Barrera L. (2017). El bienestar psicológico y la satisfacción con la vida como predictores del bienestar social en una muestra de universitarios ecuatorianos. Salud & Sociedad.

[B49-ejihpe-15-00088] Orellana E., Segovia J. (2014). Evaluación del clima social escolar mediante semilleros de convivencia de los octavos de educación general básica. Tesis de pregrado.

[B50-ejihpe-15-00088] Oyanedel J. C., Alfaro J., Mella C. (2015). Bienestar subjetivo y calidad de vida en la infancia en Chile. Revista Latinoamericana de Ciencias Sociales, Niñez y Juventud.

[B51-ejihpe-15-00088] Reicher S., Emler N. (1985). Delinquent behaviour and attitudes to formal authority. British Journal of Social Psychology.

[B52-ejihpe-15-00088] Rodríguez-Garcés C., Espinosa D., Padilla G. (2019). Sense of belonging at school among children and adolescents in Chile: Profiles and paths through decision tree. Revista Colombiana de Educación.

[B53-ejihpe-15-00088] Rodríguez-Manteca F. A., Rodríguez-Bravo A. E. (2024). Etiología y metodología de intervención en los procesos de fracaso y abandono escolar. Una revisión sistemática y metaanálisis. Revista Complutense de Educación.

[B54-ejihpe-15-00088] Ruvalcaba N., Gallegos J., Fuerte J. M. (2017). Competencias socioemocionales como predictoras de conductas prosociales y clima escolar positivo en adolescentes. Revista Interuniversitaria de Formación del Profesorado.

[B55-ejihpe-15-00088] Salmela K., Tuominen H. (2010). Adolescents life satisfaction during the transition to post-comprehensive education: Antecedents and consequences. Journal of Happiness Studies.

[B56-ejihpe-15-00088] Sanz R., López-Luján E., Serrano A., Giménez-Beut J. A. (2023). Emotional competences of primary education teachers: A need in school post COVID-19. European Journal of Investigation in Health, Psychology and Education.

[B57-ejihpe-15-00088] Schumacker R. E., Lomax R. G. (2004). A beginner’s guide to structural equation modeling.

[B58-ejihpe-15-00088] Serrano A., Sanz R., Cabanillas J. L., López-Lujan E. (2023). Socio-emotional competencies required by school counsellors to manage disruptive behaviours in secondary schools. Children.

[B59-ejihpe-15-00088] Smith T. E., Holmes S. R., Romero M. E., Sheridan S. M. (2022). Evaluating the effects of family-school engagement interventions on parent-teacher relationships: A meta-analysis. School Mental Health.

[B60-ejihpe-15-00088] Spady W. (1971). Dropouts from higher education: Toward an empirical model. Interchange.

[B61-ejihpe-15-00088] Steffgen G., Recchia S., Viechtbaueer W. (2013). The link betweeen school climate and violence in school: A meta-analytic review. Aggresion and Violent Behavior.

[B62-ejihpe-15-00088] Steinmayr R., Spinath B. (2009). The importance of motivation as a predictor of school achievement. Learning and Individual Differences.

[B63-ejihpe-15-00088] Tinto V. (1975). Dropout education: A theoretical synthesis of recent research. Review of Educational Research.

[B64-ejihpe-15-00088] Valle A., Cabanach R., Rodríguez S., Núñez J., González-Pienda J. (2006). Metas académicas, estrategias cognitivas y estrategias de autorregulación del estudio. Psicothema.

[B65-ejihpe-15-00088] Veas A., López-López J. A., Gilar-Corbi R., Miñano P., Castejón J. L. (2017). Differences in cognitive, motivational and contextual variables between under-achieving, normally-achieving, and over-achieving Spanish students: A mixed-effects analysis. Psicothema.

[B66-ejihpe-15-00088] Ventura-León J., Caycho T., Talledo K. (2021). Satisfacción académica en estudiantes de Ciencias de la Salud antes y durante la pandemia COVID-19. Revista Habanera de Ciencias Médicas.

[B67-ejihpe-15-00088] Vera A., Cerda G., Aragón E., Pérez C. (2021). Rendimiento académico y su relación con las variables socioemocionales en estudiantes chilenos de contextos vulnerables. Educación XX1.

[B68-ejihpe-15-00088] Villalobos B., Carrasco C., Olavarría D., Ortiz S., López V., Oyarzún D., Ascorra P., Ayala A., Bilbao A., Morales M., Álvarez J. P. (2016). Victimización de pares y satisfacción con la vida: La influencia del apoyo de profesores y compañeros de la escuela. Psykhe.

[B69-ejihpe-15-00088] Weiner B. (1986). An attributional theory of motivation and emotion.

[B70-ejihpe-15-00088] Yulianti K., Denessen E., Droop M., Veerman G. J. (2022). School efforts to promote parental involvement: The contributions of school leaders and teachers. Educational Studies.

